# Prenatal Diagnosis of Pulmonary Atresia With Ventricular Septal Defect and an Aberrant Ductus Arteriosus in a Dextrocardia by Two- and Three-Dimensional Echocardiography: A Case Report

**DOI:** 10.3389/fmed.2022.904662

**Published:** 2022-07-01

**Authors:** Lulu Liang, Yu Wang, Ying Zhang

**Affiliations:** Department of Ultrasound, Shengjing Hospital of China Medical University, Shenyang, China

**Keywords:** fetus, pulmonary atresia with ventricular septal defect, aberrant ductus arteriosus, dextrocardia, 3D

## Abstract

**Introduction:**

Prenatal diagnosis of pulmonary atresia is difficult in relative, especially when the pulmonary artery is slim and hypoplastic in development. It is of great importance to search for the blood supply to the pulmonary artery in those fetuses while it challenges most screening sonographers, even fetal echocardiography specialists. We herein report a rare case of pulmonary atresia with ventricular septal defect, complicated with an aberrant ductus arteriosus which provides the blood supply to the pulmonary artery. Besides, the case was also accompanied by cardiac malposition, dextrocardia with situs solitus. The echocardiographic characteristics and autopsy findings are also presented to approach the skill of fetal diagnosis.

**Case presentation:**

A 30-year-old primigravida woman was referred to our center at gestational age of (24 ± 3) weeks for further fetal cardiac examination for suspected fetal cardiac anomalies. Fetal echocardiography revealed dextrocardia, situs solitus of the atria, an L-ventricular loop, a ventricular septal defect, an enlarged coronary sinus, and pulmonary atresia by transverse scanning. The ductus arteriosus was not present at the three-vessel trachea view with the retrograde flow showing in the pulmonary artery trunk, which suggested the possibility of an aberrant ductus arteriosus. Sagittal and coronal scanning was attempted to find that the pulmonary artery connected with the innominate artery *via* the aberrant ductus arteriosus. Three-dimensional echocardiography with spatio-temporal image correlation and high-definition flow imaging technique was performed to obtain the three-dimensional rendered image, which clearly showed the malformation in space. The pregnancy was terminated and the gross findings confirmed the prenatal diagnosis.

**Conclusion:**

A detailed evaluation of fetal cardiac anatomy and hemodynamics is crucial for the detection of an aberrant ductus arteriosus, which plays an important role in the diagnosis of pulmonary atresia with ventricular septal defect. Sagittal and coronal scanning is useful to find the course of this aberrant ductus arteriosus. The three-dimensional echocardiography with spatio-temporal image correlation technique could provide additional spatial information to show great arteries in detail, which can serve as a supplement to traditional two-dimensional modality and benefit examiners to make an accurate diagnosis.

## Introduction

Pulmonary atresia with ventricular septal defect (PA-VSD) is a rare congenital heart disease (CHD) with an incidence of 7 per 100,000 live births (1). Pulmonary atresia is defined as the lack of luminal continuity and absence of blood flow from a ventricle or a rudimentary chamber and the pulmonary artery (2). It is difficult to make an accurate diagnosis of pulmonary atresia prenatally, especially when the pulmonary artery is slim and hypoplastic. During the cardiac assessment, it is crucial to clarify the hemodynamics to identify the blood supply to the pulmonary artery. However, this challenges most examiners when an aberrant ductus arteriosus (DA) is in existence as it may not be shown in routine cardiac planes. In this study, we report a rare fetal case of PA-VSD with this “special” DA-pulmonary artery blood supplying pattern. The fetus was also accompanied by dextrocardia with situs solitus, which made the diagnosis more difficult. The strategy for the diagnosis of PA-VSD together with the experience for tracing the aberrant DA was summarized in this report. In addition, the usage of the advanced three-dimensional (3D) echocardiography with spatio-temporal image correlation (STIC) was proposed to show the spatial relationship of the great arteries for this complex malformation, which helped to reach an accurate diagnosis confirmed by postmortem findings.

## Case Presentation

**Figure 1:**
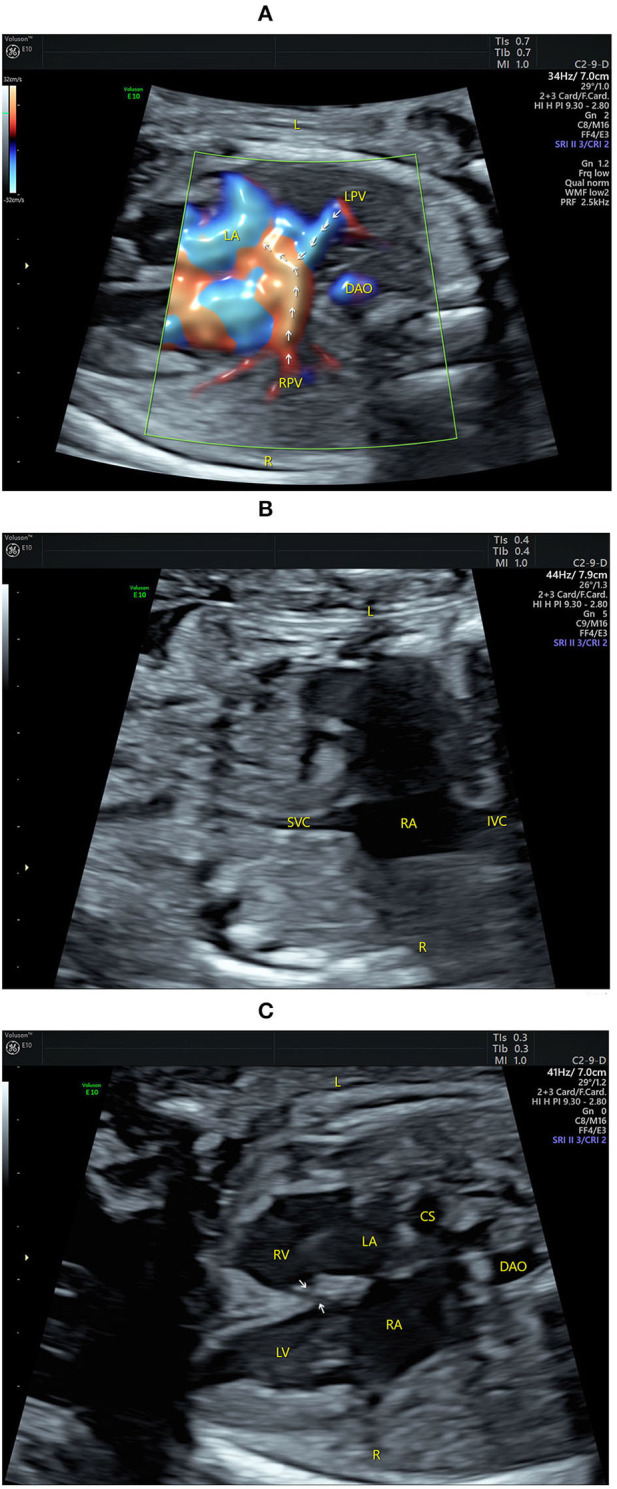
Echocardiograms showing fetal atrial situs and the four-chamber view (4CV). **(A)** The pulmonary veins drain into the left atrium (indicated by arrows) on the left of the fetus by high-definition flow imaging (HDFI). **(B)** The superior vena cava and the inferior vena cava are connected to the right atrium on the right of the fetus. **(C)** The 4CV shows a heart of normal size with balanced chambers, situs solitus of the atria, atrioventricular discordance, a VSD (indicated by arrows), and an enlarged coronary sinus. CS, coronary sinus; DAO, descending aorta; IVC, inferior vena cava; L, left; LA, left atrium; LPV, left pulmonary vein; LV, left ventricle; R, right; RA, right atrium; RPV, right pulmonary vein; RV, right ventricle; SVC, superior vena cava.

**Figure 2:**
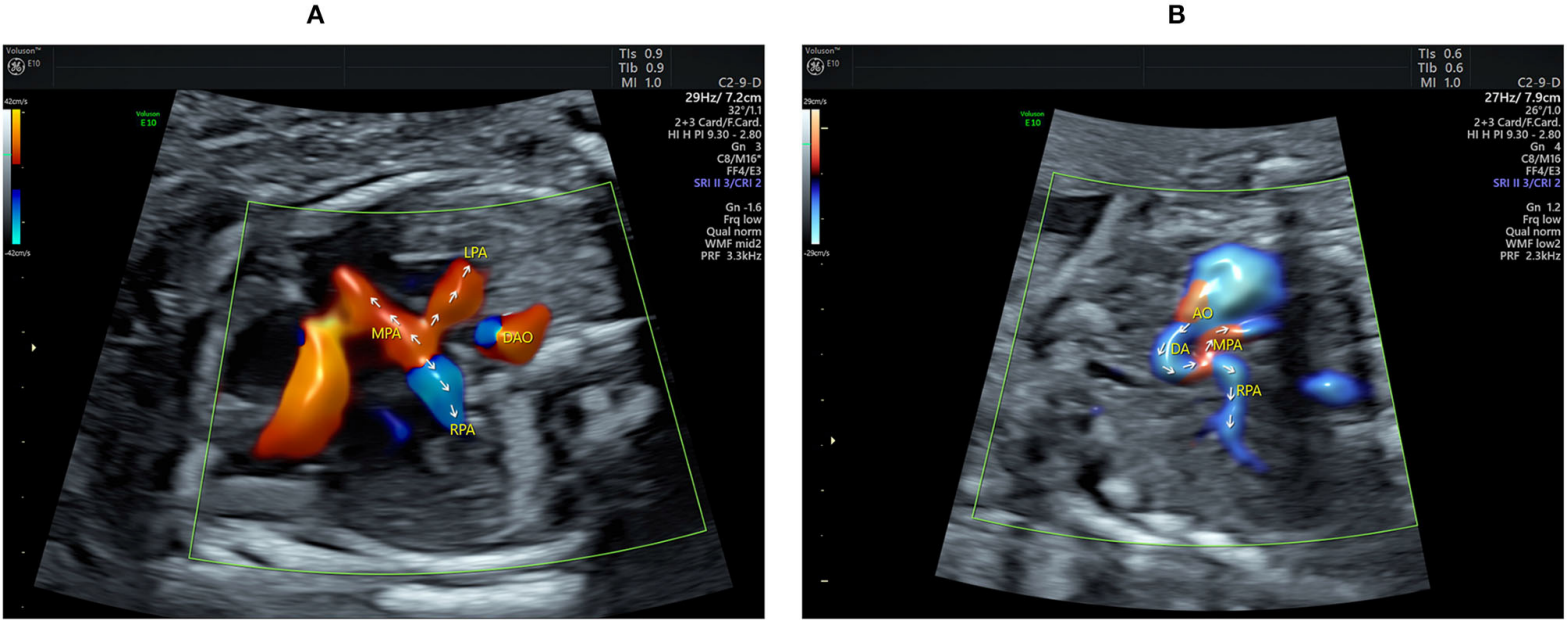
Echocardiograms showing the main pulmonary artery (MPA) with its branching and the aberrant ductus arteriosus (DA). **(A)** The Color flow mapping shows the antegrade flow in both the LPA and RPA together with the retrograde flow in the distal MPA (indicated by arrows). **(B)** The course and the direction of the blood flow (indicated by arrows) in the DA are clearly shown when using HDFI in a parasagittal view. AO, aorta; DA, ductus arteriosus; DAO, descending aorta; LPA, left pulmonary artery; MPA, main pulmonary artery; RPA, right pulmonary artery.

A 30-year-old primigravida woman was referred to our center at gestational age of (24 ± 3) weeks for further fetal cardiac examination for suspected cardiac anomalies. The patient was in good health without any maternal complications or high-risk factors (e.g., diabetes, hypertension, and amniotic disorders). A thorough examination was then performed by a fetal echocardiography specialist to assess any potential cardiac anomalies. Conventional two-dimensional (2D) echocardiography was used to show the anatomical structure of the fetus. At first, a long-axis plane of the fetus together with the transverse planes at both the fetal abdominal level and thoracic level was scanned to determine the visceral and cardiac position (3) (Figures 1A,B). The stomach was visualized on the left side while the heart in the right hemithorax with the apex pointing to the right. An additional movie file shows this in more detail (see Supplementary Video 1). A transverse scanning was then performed from the four-chamber view (4CV) up to the three-vessel trachea (3VT) view. The 4CV showed a heart of normal size with balanced chambers. The situs solitus of the atria was determined but with atrioventricular discordance and L-looped ventricles. In addition, a 6.4 mm wide VSD and an enlarged coronary sinus connecting with the left superior vena cava were also in visualization (Figure 1C). When scanning the outflow tracts planes, only a great artery was seen originating from bilateral ventricles, which was proved to be aorta as it went upward and continued to be aortic arch. To the left side of aorta, a small-sized artery was found with characteristic bifurcation. It was turned out to be the pulmonary artery while the pulmonary valve was invisible which indicated the possibility of atretic pulmonary trunk. Color Doppler provided useful information as it showed the antegrade flow in both the left and right pulmonary arteries together with the retrograde flow in the distal pulmonary trunk (Figure 2A). An additional movie file shows this in more detail (see Supplementary Video 2). At this point, the blood supply to the pulmonary artery was considered derived from the aorta, possibly through a DA. Paradoxically, no DA was found at the 3VT view. An aberrant DA was then in suspicious. A thorough scan was performed to look for the DA, if existent. A coronal scan sectioned through the bilateral hilum showed the course of the left and right pulmonary arteries while a vessel was detected connecting the confluence of the bilateral pulmonary arteries with the aorta when the sound beam turned to section through the base of an innominate artery in a parasagittal view. In fact, it represented a special ductal arch that connected the pulmonary artery with the innominate artery, instead of the descending aorta (Figure 2B). An additional movie file shows this in more detail (see Supplementary Videos 3, 4). In addition, we scanned the thymus prenatally and found no abnormalities.

**Figure 3:**
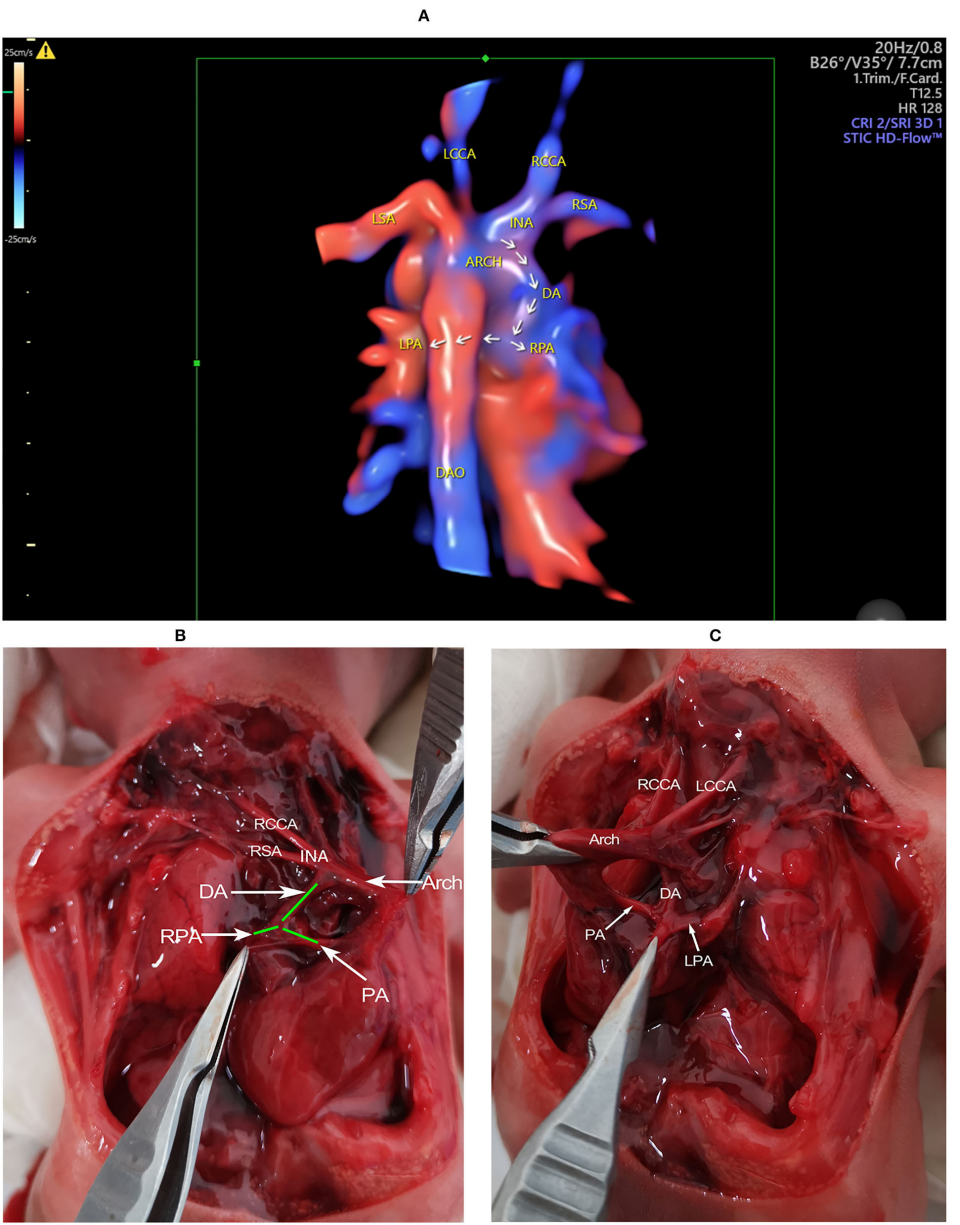
The 3D reconstructed image and gross findings. **(A)** The 3D reconstructed image showing the spatial relationship of the related great vessels. The aberrant DA connects the base of the innominate artery with the pulmonary artery. The course of the DA, the LPA, and the RPA is indicated by arrows. **(B)** The heart is in the right hemithorax with the apex pointing to the right. The aortic arch is on the left of the trachea. The DA is visualized connecting the base of innominate artery with the PA. The origination of RPA from PA is also visualized. **(C)** When pulling up the heart and the great arteries to the right side, the connection of DA with PA, together with the origination of LPA is determined. ARCH, aortic arch; DA, ductus arteriosus; DAO, descending aorta; INA, innominate artery; LCCA, left common carotid artery; LPA, left pulmonary artery; LSA, left subclavian artery; PA, pulmonary artery; RCCA, right common carotid artery; RPA, right pulmonary artery; RSA, right subclavian artery.

The 3D echocardiography together with the STIC technique was then used to obtain the 3D image of the great arteries. A 3D motorized transducer (4–8 MHz) was used to acquire cardiac volumes when scanning the coronal planes using high-definition flow imaging (HDFI). The acquisition time was set to 12.5 s and the sweep angle was set to 30°. Cardiac volumes were acquired automatically and then reconstructed to display in a cine loop in multiplanar mode showing three orthogonal planes simultaneously. Volume post-analysis was then performed using an off-line software (4D viewer, version 14.0) to obtain the 3D reconstructed images, by adjusting the size and direction of the region of interest properly and rotating three orthogonal planes, together with smooth surface and gradient light algorithm. The 3D color-rendered image can demonstrate the spatial relationship of the related great arteries, including the course and connection of the aberrant DA (Figure 3A).

The patient refused a chromosomal examination but decided to terminate the pregnancy due to a bad prognosis (Figures 3B,C).

## Discussion And Conclusion

Cardiac malposition is often an integral part of complex associated abnormalities of visceral and atrial situs. Due to the complex nature of these groups of anomalies, the segmental approach is necessary to make an accurate diagnosis. Dextrocardia has been defined classically and most consistently as the location of the heart in the right hemithorax with the apex pointing (base–apex axis) to the right (4). According to visceral and atrial situs, dextrocardia can be divided into three types: dextrocardia with situs solitus (also known as isolated dextrocardia); dextrocardia with situs inversus; and dextrocardia with situs ambiguous. Most of the previous publications (5–9) had indicated that the first type is the most common, while the first type and the last type are more likely to be associated with other cardiac abnormalities. Dextrocardia with situs solitus and atrioventricular discordance was defined as mixed dextrocardia in the study of Lev et al. (10). Different from mirror-image dextrocardia (situs inversus and atrioventricular concordance, usually with a right-sided DA), we herein reported a special case of mixed dextrocardia which complicated with PA-VSD and an aberrant DA.

It is challenging for the examiner to reach the diagnosis of PA-VSD as it should be differentiated from persistent truncus arteriosus (PTA) (11, 12). Pulmonary atresia (2) refers to the lack of luminal continuity and absence of blood flow from a ventricle or a rudimentary chamber and the pulmonary artery. The extent of pulmonary artery atresia is quite variable in PA-VSD (4). The pulmonary valve and the proximal portion of the pulmonary trunk may be involved while the distal trunk and the bilateral pulmonary arteries keeping free communication. Or more severely, the pulmonary artery bifurcation is involved, too. Rarely, only the pulmonary valve is imperforate. PTA is characterized by a single great artery arising from the base of both ventricles *via* only a single semilunar valve, which supplies the systemic, coronary, and pulmonary circulation, and by a VSD (13, 14). According to different origins of the pulmonary trunk/unilateral pulmonary artery, PTA could be classified into 3 types by Edwards (15). When differentiating PA-VSD from PTA, several characteristics should be taken into consideration during the fetal diagnosis.

- The source of blood supply to the pulmonary artery. Usually, the DA provides the blood supply to the lung in most PA-VSD fetuses when confluent pulmonary arteries are present, resulting in retrograde flow in the short pulmonary trunk together with antegrade flow in bilateral pulmonary arteries. In contrast, antegrade flow is present in the full course of the pulmonary artery, including the short trunk in type I PTA.
- The overriding degree of the great artery. Although equal biventricular origin could occur, the aorta usually arises from the right ventricle for PA-VSD (11, 12, 16). For PTA, the truncal root originates predominantly from the morphological right ventricle (11, 12, 14).
- Morphology and function of the semilunar valves (13, 14, 17). In PTA fetuses, the number of leaflets of the semilunar valve may vary from 2 to 5 and valve stenosis and/or insufficiency may present while the aortic valve is usually normal in PA-VSD.
- The presence of a short pulmonary trunk with branching. This may occur in both PA-VSD and PTA (type I).
- The distance between the semilunar valve annulus and the site bilateral pulmonary arteries given off. A larger distance is present in PA-VSD while PTA fetuses (type II/III) show a smaller distance in contrast.

In fact, it is hard to delineate all the anatomical abnormalities in the current case study. The transverse, coronal, and the parasagittal planes were scanned and obtained to show corresponding cardiac structures. Hemodynamics were analyzed to seek for the source of the blood supply to the pulmonary artery and to differentiate PA-VSD from PTA. It should be mentioned that the coronal plane sectioned through bilateral hilum is useful to find any systemic-to-pulmonary collateral arteries, when present. They may arise from descending aorta, varying from 1 to 6 in number. When the pulmonary arteries are confluent and not extremely hypoplastic, it is generally ductal source of pulmonary artery supply. However, multiple collateral arteries may present when the pulmonary arteries are non-confluent or extremely hypoplastic. These collateral arteries may connect with the central pulmonary arteries or their branches or directly enter the hilum, the course of which might be detected in the coronal plane. As no collateral artery was determined in the current case, it suggested a ductal source of pulmonary artery supply. However, no DA was shown at the 3VT view, which indicated the possibility of an aberrant DA.

**Figure 4:**
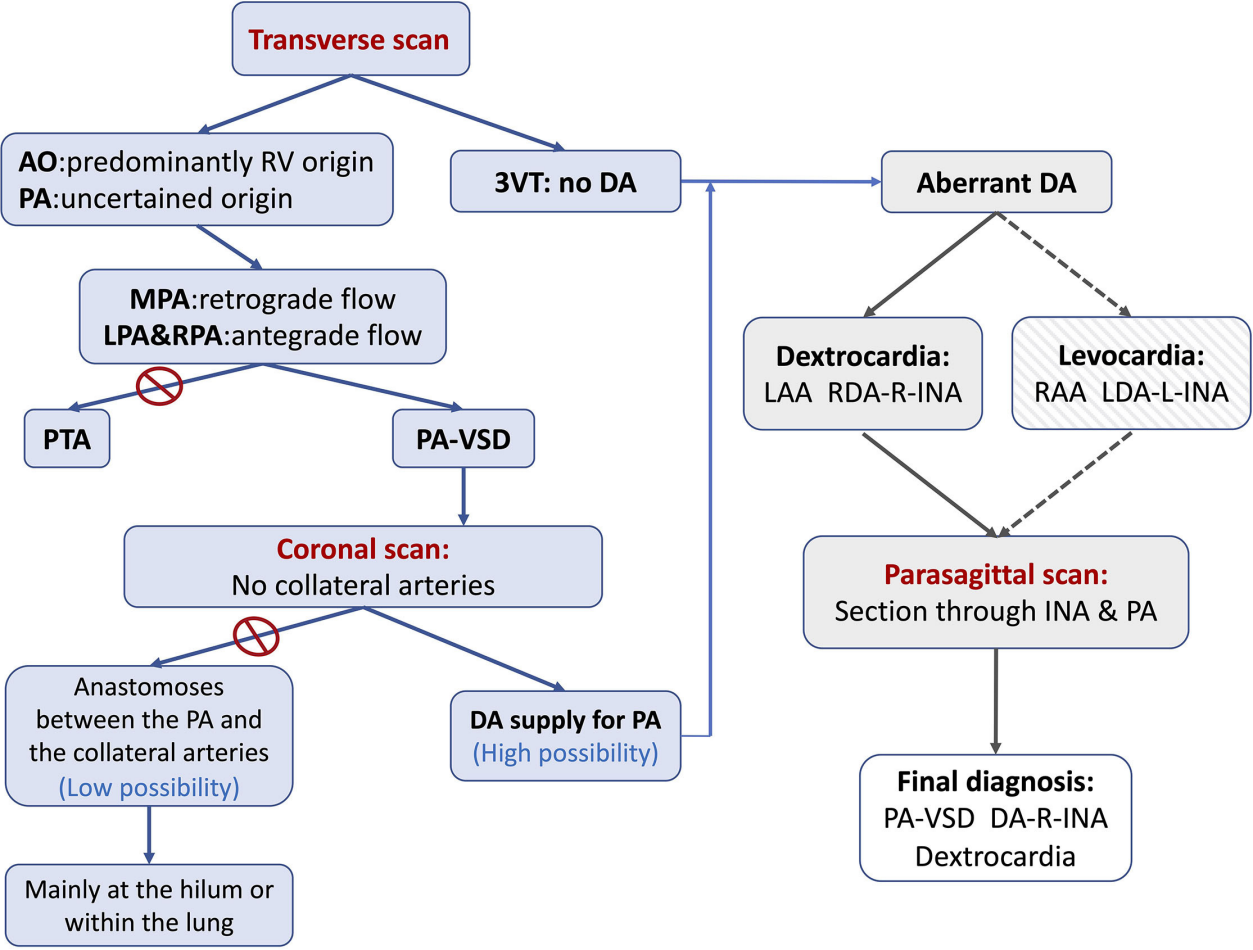
The flowchart summarizing the steps of the diagnosis of PA-VSD with an aberrant ductus in the current case study. The first step is to determine the blood supply to the pulmonary artery. The retrograde flow in MPA together with the antegrade flow in LPA and RPA suggests the DA-PA supplying pattern. The second step is to show (or rule out) any collateral artery by scanning the coronal plane sectioned through the bilateral hilum. The third step is to search for and trace the origin, course, and connection of the aberrant ductus, if existent. As the ductus may be connected to the L-INA in a mirror-imaging RAA fetus (Levocardia and RAA), there may be in existence a ductus connecting the R-INA with the pulmonary artery in the current case (Dextrocardia and LAA). An intended parasagittal scanning sectioned through the base of R-INA does the work. 3VT, three-vessel trachea view; AO, aorta; DA, ductus arteriosus; LAA, left aortic arch; LDA, left ductus arteriosus; L-INA, left innominate artery; LPA, left pulmonary artery; MPA, main pulmonary artery; PA, pulmonary artery; PA-VSD, pulmonary atresia with ventricular septal defect; PTA, persistent truncus arteriosus; RAA, right aortic arch; RDA, right ductus arteriosus; R-INA, right innominate artery; RPA, right pulmonary artery.

The previous reports (18) had indicated that conotruncal anomalies were frequently associated with right aortic arch (RAA). An aberrant DA may connect the pulmonary artery with the left innominate artery (L-INA) in the case of mirror-image branching RAA. As a right-sided heart together with a left-sided aortic arch was present in the current case, it resembles a mirror-image situation of RAA-L-INA. We speculate that there may be a DA connecting with the right-sided INA just like the aberrant DA in RAA-L-INA. According to our experience (18), a parasagittal scan is useful to detect this special DA, which could hardly be shown in transverse planes. The steps of the diagnosis were summarized in a flowchart (Figure 4).

New color techniques, including radiant flow (R-flow) imaging, were used in the current case study. This technology allows the index of erythrocyte density in a certain area converting into a height index by a specific algorithm and then superimposing on the initial coding of color (e.g., color Doppler and HD flow). Thus, the flow is displayed with a sense of depth, reducing blood overflow, and indicating the vessel with sharper edges than is possible with color alone (19). The blood flow showed by CDFI-R-flow in the pulmonary trunk and its branches and the ductus greatly enhanced the morphological information and impressively showed the course of these vessels. In addition, fetal cardiac volumes with HDFI were acquired and post-analyzed using STIC to obtain the 3D color-rendered image which can provide additional information of flowing blood and anatomical details of the great vessels (18). These vessels were shown in a three-dimensional perspective in a clear and impressive image. The novel 3D technology provides new insights and has the potential to supplement traditional 2D echocardiography by yielding realistic-like images, which may contribute to prenatal counseling between obstetricians and parents.

As a conotruncal cardiac abnormality, the PA-VSD was frequently accompanied by additional intracardiac, extracardiac, and chromosome abnormalities, especially 22q11 microdeletions (11, 16). In the study of Gottschalk et al. (20), 38% of the 50 cases with prenatal diagnosis of PA-VSD were associated with chromosome abnormalities, and 22q11 microdeletions accounted for 68% of them. Investigators (21) have suggested that 22q11 microdeletion is more likely to be complicated with absence of DA, hypoplasia, or absence of main pulmonary artery, and complex source of pulmonary blood supply. Therefore, we strongly recommend that chromosomal tests should be performed for these fetuses.

In summary, we herein present a rare case of PA-VSD with an aberrant right DA providing the blood supply to the pulmonary arteries in a dextrocardia, for the first time. The diagnostic approaches and differential diagnosis strategies of PA-VSD are discussed. As a supplement to 2D echocardiography, the 3D-rendered images can provide additional effective information and be helpful to understand and observe the course of an aberrant DA.

## Data Availability Statement

The original contributions presented in the study are included in the article/Supplementary Material, further inquiries can be directed to the corresponding authors.

## Ethics Statement

The studies involving human participants were reviewed and approved by the Ethics Committee of Shengjing Hospital of China Medical University. The patients/participants provided their written informed consent to participate in this study. Written informed consent was obtained from the individual(s) for the publication of any potentially identifiable images or data included in this article.

## Author Contributions

LL drafted the manuscript. YZ performed the fetal echocardiography. YW performed the 3D post-analysis of the cardiac volumes. All authors read and approved the final manuscript.

## Funding

This case study was funded by the 345 Talent Project of Shengjing Hospital. The funders had no role in study design, data collection and analysis, decision to publish, or preparation of the manuscript.

## Conflict of Interest

The authors declare that the research was conducted in the absence of any commercial or financial relationships that could be construed as a potential conflict of interest.

## Publisher's Note

All claims expressed in this article are solely those of the authors and do not necessarily represent those of their affiliated organizations, or those of the publisher, the editors and the reviewers. Any product that may be evaluated in this article, or claim that may be made by its manufacturer, is not guaranteed or endorsed by the publisher.

## Supplementary Material

Supplementary Video 1 Echocardiograms determining the fetal visceral and cardiac position. Scan the long-axis plane of the fetus to show the fetal head position and posture and then rotate the sound beam 90° counterclockwise to show the transverse planes at both the fetal abdominal level and thoracic level. The gastric vacuole is visualized on the left side while the heart in the right hemithorax with the apex pointing to the right.

Supplementary Video 2 Echocardiograms showing the outflow tracts planes of the fetus. Only a great artery is seen arising from bilateral ventricles, mainly from the right ventricle. It proves to be aorta as it goes upward and continues to be aortic arch and descending aorta. To the left side of aorta, a small-sized artery is found with characteristic bifurcation, which is turned out to be the pulmonary artery. The pulmonary valve is invisible. The antegrade flow is identified in both pulmonary arteries while the retrograde flow is seen in the pulmonary trunk.

Supplementary Video 3 Echocardiograms showing the coronal plane sectioned from bilateral hilum. The left and right pulmonary arteries are visualized entering ipsilateral hilum, respectively. In addition, no collateral artery is identified arising from descending aorta.

Supplementary Video 4 Echocardiograms showing the branching patterns of aortic arch and the origin, course, and connection of the aberrant ductus. The origin of the ductus is visualized when the sound beam sections through the base of the innominate artery. When rotating the sound beam slightly and turning to the parasagittal plane, the full course of the ductus and its connection with the pulmonary artery are determined.

## Abbreviations

2D: two-dimensional
3D: three-dimensional
3VT: three-vessel trachea view
4CV: four-chamber view
CHD: congenital heart disease
DA: aberrant ductus arteriosus
HDFI: high-definition flow imaging
L-INA: left innominate artery
PA-VSD: pulmonary atresia with ventricular septal defect
PTA: persistent truncus arteriosus
RAA: right aortic arch
R-flow: radiant flow
STIC: spatio-temporal image correlation.
